# The Latest Succession of Dinosaur Tracksites in Europe: Hadrosaur Ichnology, Track Production and Palaeoenvironments

**DOI:** 10.1371/journal.pone.0072579

**Published:** 2013-09-03

**Authors:** Bernat Vila, Oriol Oms, Víctor Fondevilla, Rodrigo Gaete, Àngel Galobart, Violeta Riera, José Ignacio Canudo

**Affiliations:** 1 Grupo Aragosaurus–IUCA, Paleontología, Facultad de Ciencias Universidad de Zaragoza, Zaragoza, Spain; 2 Institut Català de Paleontologia Miquel Crusafont, Sabadell, Barcelona, Spain; 3 Departament de Geologia (Estratigrafia), Facultat de Ciències Universitat Autònoma de Barcelona, Cerdanyola del Vallès, Barcelona, Spain; 4 Museu de la Conca Dellà, Isona i Conca Dellà, Lleida, Spain; University of Birmingham, United Kingdom

## Abstract

A comprehensive review and study of the rich dinosaur track record of the Tremp Formation in the southern Pyrenees of Spain (Southwestern Europe) shows a unique succession of footprint localities prior to the end-Cretaceous mass extinction event. A description of some 30 new tracksites and data on sedimentary environments, track occurrence and preservation, ichnology and chronostratigraphy are provided. These new track localities represent various facies types within a diverse set of fluvial environments. The footprint discoveries mostly represent hadrosaurian and, less abundantly, to sauropod dinosaurs. The hadrosaur tracks are significantly smaller in size than, but morphologically similar to, those of North America and Asia and are attributable to the ichnogenus *Hadrosauropodus*. The track succession, with more than 40 distinct track levels, indicates that hadrosaur footprints in the Ibero-Armorican region occur predominantly in the late Maaastrichtian (at least above the early Maastrichtian–late Maastrichtian boundary). The highest abundance is found noticeably found in the late Maastrichtian, with tracks occurring in the C29r magnetochron, within about the latest 300,000 years of the Cretaceous.

## Introduction

The end-Cretaceous mass extinction was one of the major events in the history of life on Earth, resulting in the demise of multiple taxa [1]. On land, non-avian dinosaurs and many other vertebrates succumbed at the end of the Maastrichtian, the final stage of the Cretaceous. With the exception of data from the bolide impact zone and nearby areas (e.g. North America [2]), little is known about how the last dinosaur faunas reached the boundary in most parts of the planet [3]–[7]. Much of the current knowledge is from the fossil bone record recovered from the uppermost levels of many geologic formations around the world. In addition, the discovery of dinosaur tracks close to the Cretaceous–Palaeogene (K–Pg) boundary has shown their utility as chronostratigraphical markers [8]. The autochthonous character of fossil tracks means that they provide unmistakable proof of the presence of the track maker in a restricted temporal and spatial context, with no possibilities of reworking as is possible for bone remains. Tracks thus represent a valuable tool for analysing last occurrences and diversity patterns of dinosaurs before the K–Pg extinction event.

Geologic formations of Campanian and Maastrichtian age all over the world provide a rich track record of dinosaurs in the last 20 million of years of the Mesozoic [9]. Up until now the geologically uppermost known track record has been located in North America, more specifically within the Raton Formation of Colorado, where a diverse ichnofauna composed of ichnites from hadrosaurs, probable ceratopsians and large theropods has been identified very close to the K–Pg boundary [10]. Nevertheless, in the last decade new discoveries in other regions have brought to light an ichnological record comparable with that of North America in terms of age and stratigraphic position [11], [12]. The Tremp Formation in the southern Pyrenees preserves one of the richest terrestrial track records yet identified in the latest Cretaceous of Europe. The dinosaur track record is composed of multiple footprint localities of Maastrichtian age with abundant tracks and trackways made by titanosaurian sauropods (Fumanya, Orcau-2, La Massana localities; [13]–[16]) as well as of hadrosaurian ornithopods (La Mata del Viudà, Moror B, Areny 1 localities; [17]–[19]) and theropods (Moror A locality; [18]). Other reports of purported dinosaur tracks are herein considered too poorly preserved to be of ichnotaxonomic significance (Mas Morull, Santa Maria de Meià, Coll de Jou localities; [20]–[22]) or of non-dinosaurian affinity (La Posa locality; [23]). Outside of the Pyrenees, Herrero-Santos [24] reported a hadrosaur trackway from the lower Maastrichtian deposits of Sierra de los Gavilanes (Murcia province, Spain), and Gierlinski et al. [25] reported hadrosaur and theropod tracks from an upper Maastrichtian locality in Poland.

The aim of the present paper is to provide the first comprehensive review and update of the latest Cretaceous dinosaur track record in Europe with the inclusion of 28 new localities, and to discuss their implications in terms of ichnotaxonomy, palaeoenvironments, chronostratigraphy, and the K–Pg boundary extinction event.

### Geological setting

**Figure 1:**
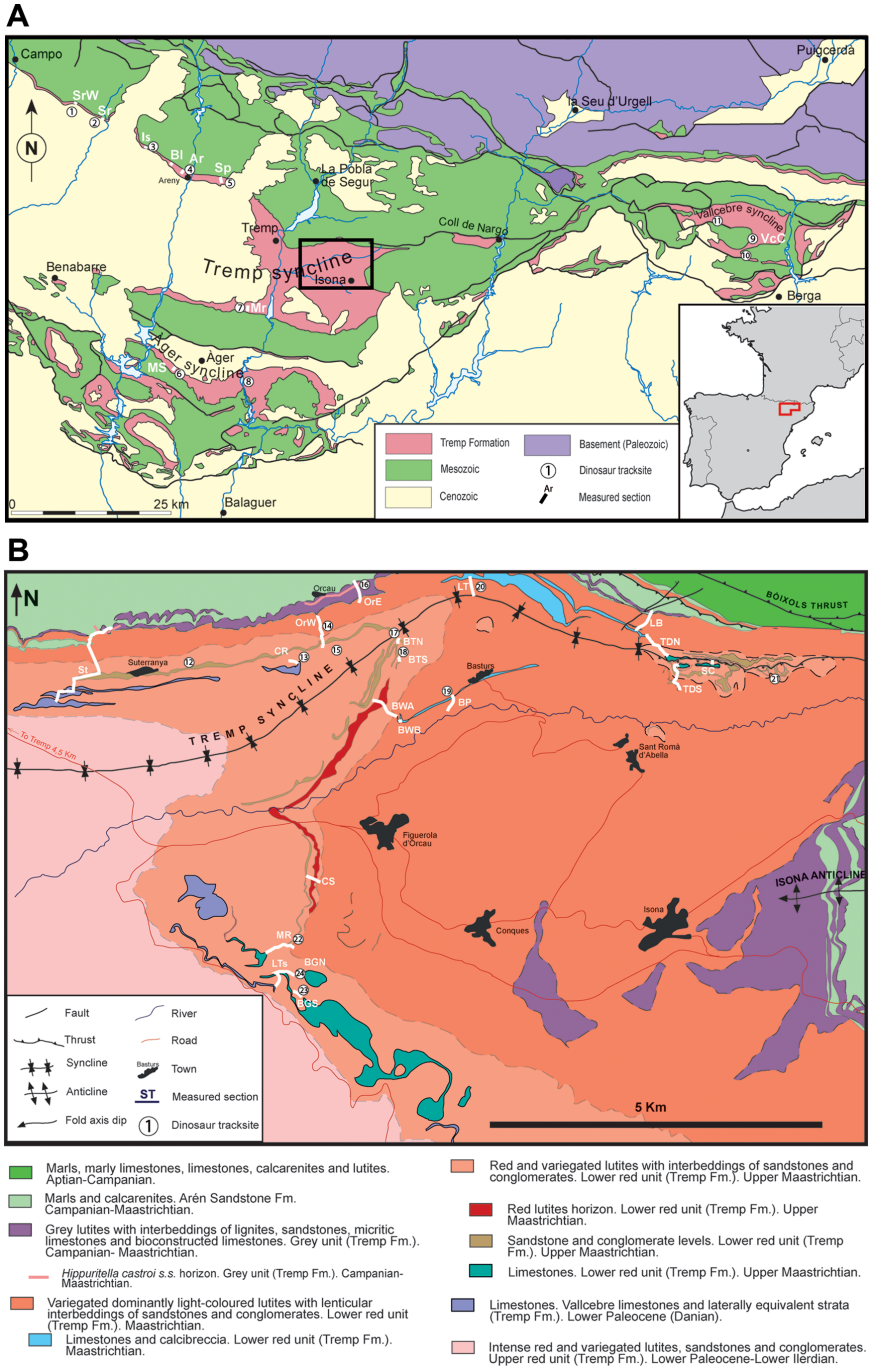
Geographic and geological setting of the study area. (A) Geological map of the southern Pyrenees with Tremp Formation outcrops and location of tracksites and measured sections (modified from López-Martínez and Vicens [26]). (B) Geological map of the Isona sector (enlarged area in A) and location of tracksites and measured sections (modified from Riera et al. [6]). Numbers (tracksites): 1, Fornons 3 and Dolor 2; 2, Serraduy Norte and Serraduy Sur; 3, Iscles-1, Iscles-2, Iscles-3, Iscles-4, and Iscles-5; 4, Areny 1; 5, Sapeira-1 and Sapeira-2; 6, La Mata del Viudà; 7, Moror A and Moror B; 8, La Massana; 9, Fumanya; 10, Cingles del Boixader; 11, La Pleta Resclosa and La Pleta Nord; 12, Camí de les Planes and Serrat de Santó; 13, Costa Roia; 14, Torrent de Carant; 15, Serrat de Sanguin; 16, Orcau-2; 17, Orcau-4; 18, Barranc de Torrebilles-5; 19, Basturs Poble; 20, La Llau de la Costa; 21, Tossal del Gassó; 22, Masia de Ramon Petjades; 23, Barranc de Guixers-1 and Barranc de Guixers-2; 24, Barranc de Guixers-3. See abbreviations for measured sections in ”Methodology” section.

The study area is concentrated on several localities belonging to the Tremp Formation, along multiple sections distributed over various geographical areas of the southern Pyrenees (Tremp, Àger, and Vallcebre synclines in the provinces of Huesca, Lleida and Barcelona, Spain, SW Europe; Fig. 1). The Tremp Formation is a marginal marine and terrestrial unit, about 800 m thick, which is exposed in northern Catalonia and Aragón (Spain) and encompasses deposits of Late Cretaceous to Early Palaeogene age. The Cretaceous (Maastrichtian) part of the formation contains two lithologic units deposited as a result of a marine regression [27]: a basal lagoonal grey unit (coals, mudstones and sandstones) and a fluvial lower red unit (mudstones and sandstones) [28]. In the latter unit various lithostratigraphic subunits have been recognised such as the fluvial “Gres à reptiles” and the lacustrine “Tossal de la Doba limestones” (or “Tossal d'Obà” member), in the Vallcebre and Isona sectors, respectively [6], [28]–[31]. The lacustrine Vallcebre limestones and laterally equivalent strata (the “Suterranya limestones” and “Sant Salvador de Toló limestones” subunits in the Isona sector) and overlying fluvial units represent the Palaeogene strata [28], [29]. The transition from Cretaceous to Palaeogene strata is isochronous [32]. Even though no impact layer has ever been found in the Pyrenean continental sections, the Cretaceous–Palaeogene boundary is located at the contact between the lower red unit and the Vallcebre limestones and laterally equivalent strata or just below this contact, according to biostratigraphic and magnetostratigraphic determinations ([6], [31] and references therein). In any case the boundary is found above the “Gres à reptiles” and “Tossal de la Doba limestones” members. With regard to the base of the Tremp Formation, this is not completely isochronous since laterally it evolves into the deltaic-marine Arén Sandstone Formation (Fig. 3 in [6]). This chronostratigraphical scheme can be expanded and correlated to other areas of the northern Pyrenees and Provence regions of France, within what is known as the Ibero-Armorican domain [33], though no dinosaur tracks have yet been reported there. Since the early 1920 s numerous fossil localities in the Arén Sandstone and Tremp formations of Spain have yielded multiple bones, tracks and eggs attributed to theropods, hadrosaurs, ankylosaurians, and sauropods [6], [34].

**Figure 3:**
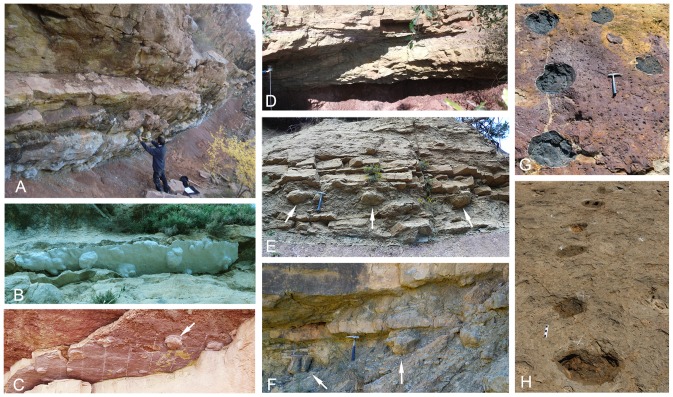
Track occurrence in the Tremp Formation. (A–D) Underneath views of overhanging ledges in the Iscles-3, Cingles del Boixader, La Mata del Viudà, and Masia de Ramon Petjades localities, respectively. (E and F) Cross-sectional outcrop views in the La Pleta Nord and Serraduy Sur localities, respectively. (G and H) Plan view outcrops in the Areny 1 and La Llau de la Costa localities, respectively. Scale bar: 15 cm; hammer length is about 33 cm. Arrows indicate the position of some tracks.

## Methods

### Abbreviations

MCD: Museu de la Conca Dellà; MPZ: Museo Paleontológico de Zaragoza; IPS: Institut Català de Paleontologia Miquel Crusafont; TL: track length; TW: track width; SL: stride length; VcC: Vallcebre composite; IsC: Isona composite; SC: Serrat del Corb; TDS: Tossal de la Doba Sud; TDN: Tossal de la Doba Nord; LB: Lo Bas; LT: Les Torres; BP: Basturs Poble; BWB: Basturs West B; BWA: Basturs West A; BTN: Barranc de Torrebilles Nord; BTS: Barranc de Torrebilles Sud; OrE: Orcau Est; OrW: Orcau Oest; BGS: Barranc de Guixers Sud; CS: Costa de la Serra; BGN: Barranc de Guixers Nord; LTs: Lo Tossal; Mr: Moror; MR: Masia de Ramon; CR: Costa Roia; St: Suterranya; Sp: Sapeira; Ar: Areny; Bl: Blasi; Is: Iscles; Sr; Serraduy; SrW; Serraduy West; MS: Mas de Saurí.

### Field data acquisition

All the localities and stratigraphic sections are indicated in Figure 1. They have been correlated with the lithostratigraphy and integrated in a chronostratigraphic frame. The western and eastern sectors of the Tremp Syncline (Isona and Isàvena-Ribagorçana areas) have been correlated and dated in accordance with data from the present study and the magnetostratigraphy of Oms and Canudo [35], Pereda-Suberbiola et al. [36], and Cruzado-Caballero et al. [37], and Riera et al. [6], Marmi et al. [38], and Vila et al. [39], respectively. The Vallcebre sector is dated on the basis of the magnetostratigraphy of Oms et al. [31] and Vila et al. [40]. The Àger sector is correlated and dated on the basis of López-Martínez et al. [20]. Their magnetostratigraphic succession for this area fits well in the standard time scale of Ogg and Hinnov [41] and Renne et al. [42], allowing correlation within the four sectors. Sections St, MR, LTs, BGS, CS, OrE, BWA, BP, LT, LB, TDN, TDS and SC correspond to sections I, V, VI, VII, IX, XIII, XIV, XVII, XVIII, XXII, XX, XXV and XXVII in Riera et al. [6], respectively. Sections Bl, CR, Mr and BWB correspond to sections H2, I1B, M1 and I15B in Riera [43], respectively. Sections BTS and BTN correspond to sections A and B in Marmi et al. [38]. Sections Ar, Sr, IsC and VcC have been redrawn from Vila et al. [39]. Sections MS and SrW have been redrawn from Llompart [17] and López-Martínez et al. [20], and Cruzado-Caballero et al. [37], respectively. The Areny 1, Tossal del Gassó, Camí de les Planes, Serrat de Santó, Orcau-4, Serrat de Sanguin, La Pleta Nord, La Pleta Resclosa, and Serraduy Norte sites are projected at the reference sections.

Detailed sedimentological analyses were conducted at Iscles-3, Masia de Ramon Petjades, Costa Roia, Serraduy Sur, La Llau de la Costa, Cingles del Boixader and La Mata del Viudà localities. High resolution, close-range photogrammetric models for MCD-5140 and MCD-5142 tracks in the La Llau de la Costa locality were generated using the methods described by Falkingham [44] in order to produce higher fidelity models (Appendix S1). Measurements of tracks and trackways refer to the parameters TL, TW and SL, taken after Thulborn [45], in cm. All necessary permits were obtained for the described study, which complied with all relevant regulations. The Departament de Cultura de la Generalitat de Catalunya and Gobierno de Aragón issued the permission for the studied localities.

### Repositories

Thirty-five track casts and replicas have been collected and housed in repositories of the Museu d'Isona i Conca Dellà, Lleida, Spain (MCD-5140, MCD-5141, MCD-5142, MCD-5143, MCD-5144, MCD-5145, MCD-5146, MCD-5147, MCD-5148, MCD-5149, MCD-5150, MCD-5151, MCD-5152, MCD-5153, MCD-5154, MCD-5155, MCD-5156, MCD-5157, MCD-5158, MCD-5159, MCD-5160, MCD-5161, MCD-5162, MCD-5163, MCD-5164, MCD-5166), the Museo Paleontológico de Zaragoza, Zaragoza, Spain (MPZ 2012/831, MPZ 2012/830, MPZ 2012/829, MPZ 2012/828, MPZ 2012/826, MPZ 2012/827, MPZ 2012/833), and the Institut Català de Paleontologia Miquel Crusafont, Sabadell, Spain (IPS-63272, IPS-63661).

## Results

The following descriptions (see also Text S1) refer to the 28 newly discovered localities (Table 1), some of which have been briefly referred by previous authors [6], [21], [37], [46], and to two already known sites (La Mata del Viudà, Areny 1; [17], [19]). The descriptions are complemented with data from other localities (Fumanya, Orcau-2, and La Massana, Moror A and B; [13], [15], [16], [18]). This work further integrates data on the stratigraphic succession of sites, the sedimentary environments, track occurrence and preservation, ichnology and chronostratigraphy. The resulting dataset shows a unique succession of track localities prior to the terminal Cretaceous extinction event.

**Table 1:** Track localities and sedimentary environments of dinosaur track localities in the Tremp Formation.

Track locality	Geographic location	Stratigraphic position and Age	Sedimentary environment	Dinosaur Ichnotaxa
Masia de Ramon Petjades; Barranc de Guixers-1; Barranc de Guixers-3; Tossal del Gassó; Serrat de Santó; Costa Roia; Sapeira-1; Sapeira-2; Barranc de Torrebilles-5	Eastern Tremp Syncline (Isona sector)	Lower red unit C29r (late Maastrichtian)	Fluvial. Meandering streams, channel facies	*Hadrosauropodus*
Barranc de Guixers-2; Serrat de Sanguin; Camí de les Planes	Eastern Tremp Syncline (Isona sector)	Lower red unit C29r (late Maastrichtian)	Fluvial. Meandering streams, channel facies	*Hadrosauropodus*, sauropod tracks
La Pleta Nord; La Pleta Resclosa	Vallcebre Syncline sector	Lower red unit C29r (late Maastrichtian)	Fluvial. Meandering streams, channel facies	*Hadrosauropodus*
Cingles del Boixader	Vallcebre Syncline sector	Lower red unit C29r (late Maastrichtian)	Fluvial. Braided streams, channel facies	*Hadrosauropodus*
La Mata del Viudà	Àger Syncline sector	Lower red unit C29r (late Maastrichtian)	Fluvial. Braided streams, channel facies	*Hadrosauropodus*
Iscles-1, Iscles-2, Iscles-3, Iscles-4; Iscles-5; Serraduy Sur; Serraduy Norte; Dolor 2; Fornons 3	Western Tremp Syncline (Isàvena-Ribagorça sector)	Lower red unit C29r (late Maastrichtian)	Fluvial. Meandering streams, channel facies	*Hadrosauropodus*
Areny 1	Western Tremp Syncline (Isàvena-Ribagorça sector)	Grey unit C30n (late Maastrichtian)	Fluvial. Meandering streams, channel facies	*Hadrosauropodus*
La Llau de la Costa	Eastern Tremp Syncline (Isona sector)	Lower red unit C30n (late Maastrichtian)	Fluvial. Meandering streams, crevasse splay facies	*Hadrosauropodus*
Basturs Poble	Eastern Tremp Syncline (Isona sector)	Lower red unit C30r/C31n (late Maastrichtian)	Fluvial. Meandering streams, channel facies	*Hadrosauropodus*
Torrent de Carant; Orcau-4	Eastern Tremp Syncline (Isona sector)	Lower red unit C31r (late Maastrichtian)	Fluvial. Meandering streams, channel facies	*Hadrosauropodus*
Moror A	Eastern Tremp Syncline (Isona sector)	Grey unit C31r (early–late Maastrichtian)	Lagoon	*Hadrosauropodus*
Moror B	Eastern Tremp Syncline (Isona sector)	Grey unit C31r (early–late Maastrichtian)	Lagoon	theropod tracks
Orcau-2	Eastern Tremp Syncline (Isona sector)	Grey unit C31r (early Maastrichtian)	Lagoon	sauropod (titanosaur) tracks
Fumanya	Vallcebre Syncline sector	Grey unit C31r (early Maastrichtian)	Lagoon	sauropod (titanosaur) tracks
La Massana	Àger Syncline sector	Grey unit (late Campanian)	Lagoon	sauropod (titanosaur) tracks

Table includes new localities and data from sites previously reported in the literature. See Text S1 and Dataset S1 for further description of the localities and repositories.

### Sedimentary environments

**Figure 2:**
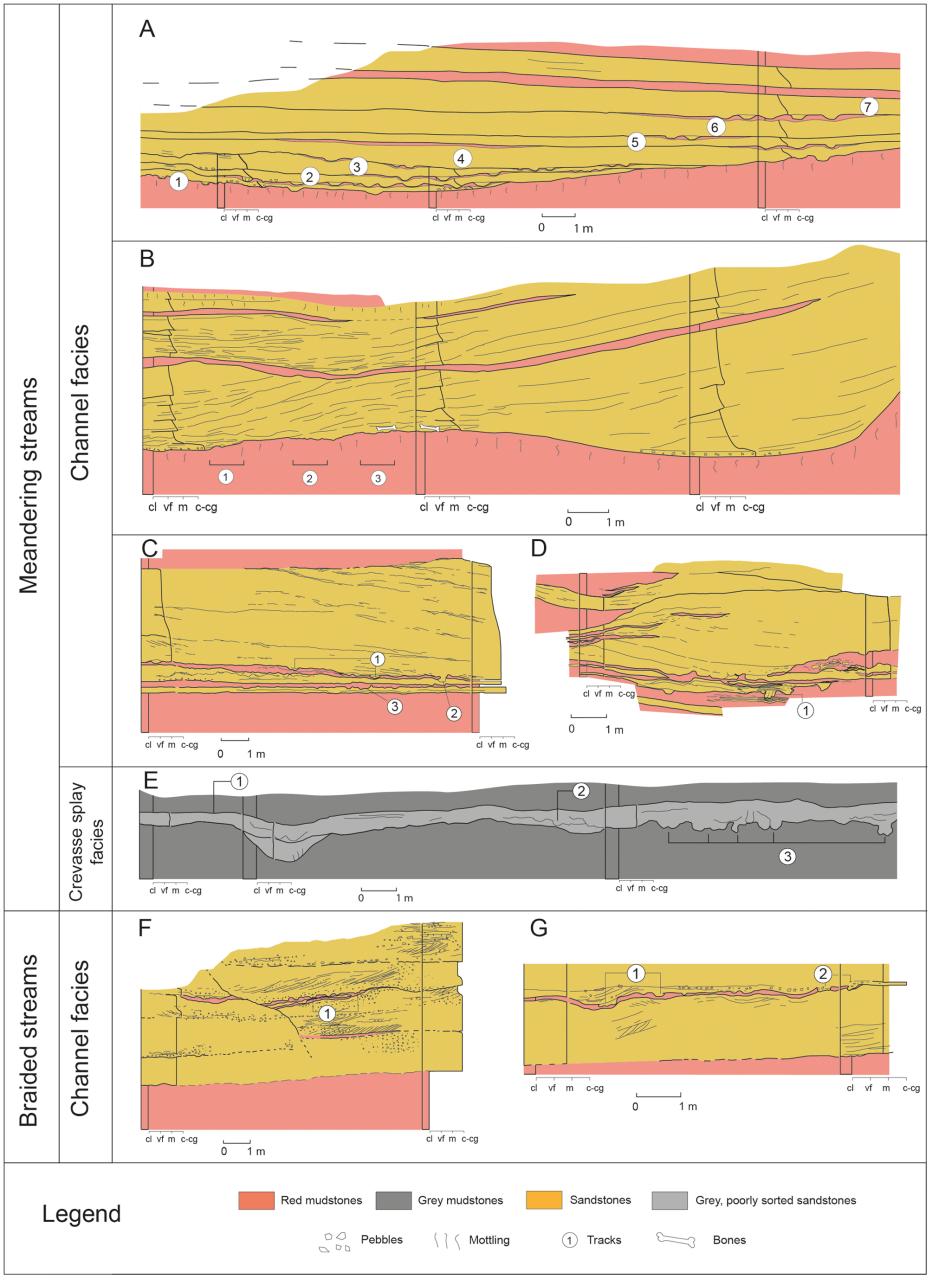
Sedimentary architecture in outcrops of the main track localities of the Tremp Formation. (A–G) Iscles-3, Masia de Ramon Petjades, Costa Roia, Serraduy Sur, La Llau de la Costa, Cingles del Boixader and La Mata del Viudà sites, respectively.

Dinosaur tracks occur in various depositional settings in the grey and lower red units of the Tremp Formation. The new sites correspond to track horizons that represent various facies types within a diverse set of fluvial environments belonging to the lower red unit (Fig. 2). The remaining localities exemplify tracks produced in lagoonal environments (Table 1).

Meandering streams (channel facies) are identified at 27 localities in the Tremp and Vallcebre synclines (Table 1) on the basis of the following characteristics (Fig. 2A–D): (a) dominant texture composed of very fine- to medium-grained sands and mudstones; (b) mudstone layers commonly covering sandstones where footprint casts are found; (c) vertical trend arranged in fining-up cycles; (d) multiple and very well-defined lateral accretions; (e) occasional conglomerate lag at the base of each accretion surface or thalweg; (f) sedimentary structures restricted to the middle and lower part of the sequence and absent in the upper part due to plant bioturbation; (g) these sedimentary structures comprise planar lamination or small-scale cross-bedding mainly visible at the top of beds; and (h) sequence thickness varying from 2 to almost 6 metres. The characteristics of the sandstone bodies correspond to lithofacies F12B of Riera et al. [6]. The channel-shape of these sandstones and the presence of the lateral accretions or point bars are typical in alluvial systems with high sinuosity rivers [47].

A crevasse splay setting can be identified at the La Llau de la Costa locality (Fig. 2E and Table 1) on the basis of the following characters: (a) poorly sorted sandstone; with (b) a tabular-shaped morphology; (c) surrounded by floodplain overbank mudstones but laterally connected to the abovementioned meandering channel bodies; and (d) an abundance of small plant remains. At the La Llau de la Costa site the bed has an exposed lateral extension of about 30 metres and a maximum thickness of 1 metre. It consists of grey, poorly sorted, fine-grained sandstones with mud and scattered coarser elements, and abundant vegetal remains, a typical feature of crevasse splay deposits [48]. Dinosaur tracks occur at the bottom of, on top of, and within the tabular lens. Hence, the multiple track levels at the La Llau de la Costa site indicate different episodes of crevasse splay development and trampling.

Braided streams (channel facies) are recognized at the localities of Cingles del Boixader and La Mata del Viudà (Vallcebre and Àger synclines, respectively; Fig. 2F and G and Table 1) on the basis of the following features: (a) general texture composed of gravels; (b) mud-free horizons except for the occasional mud drapes interbedded with sandstone layers that preserve tracks; (c) absence of a vertical trend in texture or thickness; (d) unidirectional cross-bedding in gravels; and (e) well-rounded and mineralogically mature sediment. These characteristics correspond to lithofacies 12C of Riera et al. [6].

Associated with meandering and braided streams, the fine overbank deposits consist of massive red, ochre and purple mudstones (lithofacies F10, F7 and F11 of Riera et al. [6], respectively). Invertebrate activity may also be extensive and corresponds to burrows of the continental ichnogenera *Naktodemasis* and *Spirographites* [6], which obliterate the original sedimentary structures. Grey mudstones may be present, representing oxbow-lake deposits in abandoned meanders (lithofacies F5B of Riera et al. [6]). Of particular interest in the context of dinosaur track production and preservation is the absence of mud cracking in the mudstone layers of the floodplain or within channels. When the latter are not bioturbated, they are grey-coloured and are found in the lower parts of the cycles (lithofacies F5B of Riera et al. [6]).

Lagoon settings are identified exclusively in the grey unit (Table 1) as indicated by regional works [28]. At Moror A and B, they occur in a succession of grey mudstones, marls and limestones with charophytes, root bioturbation, bivalves and ostracods [18]. The Moror A site is located in a bioturbated and bioclastic limestone bed showing evidence of desiccation, and the Moror B outcrop occurs in a micritic limestone, which is also bioturbated. Other sites in the Tremp Formation that contain sauropod footprints (Orcau-2, La Massana and Fumanya) have also been characterized as lagoonal [49], [50] with tidal influence [51]. All these lagoonal localities are found in limestones (lithofacies F2B facies of Riera et al. [6]).

### Track occurrence and preservation

Up to 28 new track localities have been identified in the Tremp Formation deposits of the southern Pyrenean basins. The track-bearing levels are very abundant and moderately extensive, especially in the Tremp syncline succession where sandstones are more abundant. It is very common to identify unmistakable footprints and track-like load structures in many levels of the lower red unit. They occur in views from below of overhanging ledges (Fig. 3A–D), in cross-sectional outcrop views (Fig. 3E, F), and more rarely in plan-view outcrops (Fig. 3G, H).

**Figure 4:**
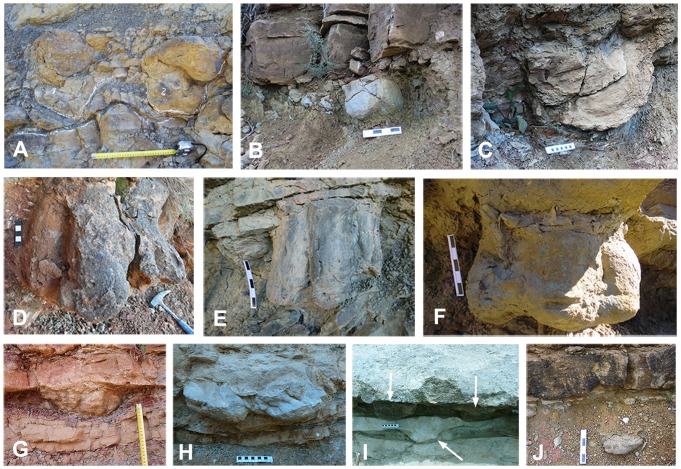
Track preservation in the Tremp Formation. (A) Footprints preserved as undetermined sandstone moulds on irregular surfaces, representing “dinoturbated” track levels. (B–E) Footprints impressed in the muddy floodplain and preserved as natural casts (convex hyporeliefs) at the base of the sandstone channel beds in the Serrat de Sanguin, La Pleta Nord, Sapeira-1, and Serraduy Sur localities, respectively. (F–I) Footprints impressed in accretion surfaces (mudstone) and preserved within the sandstone bed in the Serraduy Norte, La Pleta Nord, La Pleta Resclosa, and Cingles del Boixader localities, respectively. (J) Footprint preserved as an isolated sandstone cast within the mudstone of the floodplain in the Serrat de Sanguin locality. Numbers in A indicate tracks. Scale bars: 5 cm (in D), 10 cm (in C, H, and I), 15 cm (in E, and F), and 20 cm (in B and J); scale tape in A and G is in cm. Arrows indicate the position of some tracks.

**Figure 5:**
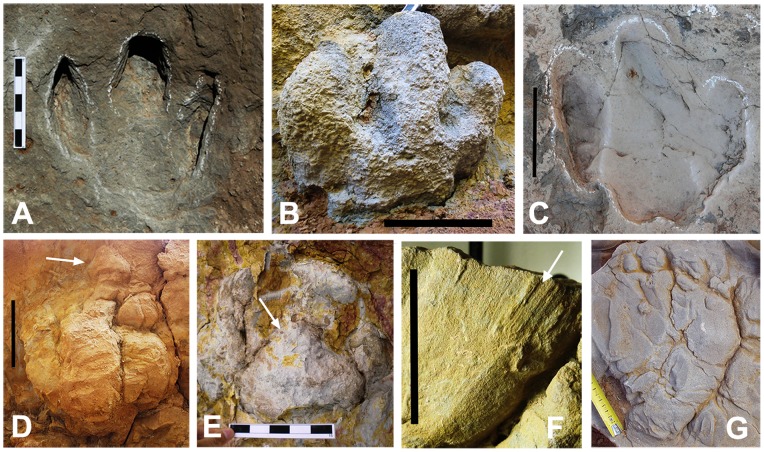
Footprint morphologies and characteristics. (A, C) Hadrosaur pedal tracks (MCD-5140 and MCD-5142, respectively) from the La Llau de la Costa locality. (B) Hadrosaur pedal track from the Iscles-3 locality. (D) Hadrosaur pedal track (uncollected) from the Barranc de Guixers-1 locality. Note the impression of the distal ungual phalanx (arrow). (E) Hadrosaur pedal track (uncollected) from the Masia de Ramon Petjades locality. Note the triangular-shaped plantar impression produced by the heel pad (arrow). (F) *Striae* or scale scratch lines (arrow) on the margins of a toe in the MPZ 2012/829 cast from the Serraduy Norte locality. (G) Sauropod pedal track (cast) from the Barranc de Guixers-2 locality. Scale bar: 15 cm (in A–E); 5 cm (in F); scale tape in G in cm.

The dinosaur tracks in the new localities are preserved in two main distinct modes: a) as natural casts (convex hyporeliefs) at the base of the sandstone beds, within the sandstone beds (i.e. in accretion surfaces), or within the mudstone levels, and b) as concave hyporeliefs on top of sandstone lenses or limestone beds. The tracks preserved as natural casts commonly occur as discrete, well-preserved moulds of footprint impressions but also as undetermined sandstone moulds on irregular surfaces, which represent “dinoturbated” track levels. Undetermined footprint casts show a globular, dish-like, rounded or ball-like shape (Fig. 4A–C) and are about 10–30 cm deep in the substrate. Well recognizable footprints reveal tridactyl, rounded or oval morphologies (Fig. 5). Some of them preserve striae or scale scratch lines on the margins of their toe or heel prints and/or slippage marks in the rear margin of the track (Fig. 5F). Some casts are three-dimensionally preserved (Fig. 4D–F) and noticeably deep (27 cm in MPZ 2012/827), revealing the complete shape of the pes or manus (MCD-5163). The sediment that fills the tracks is massive sandstone and occasionally displays burrows.

Tracks preserved as convex hyporeliefs present three modes of preservation. In mode 1 the tracks occur at the base of the sandstone bed indicating the infilling of footprints produced on the floodplain mud (Fig. 4B–E). In mode 2 the tracks occur within the sandstone bed (accretion surface) in immediate contact with the interbedded mudstones, thus reflecting infilling of tracks produced within the channel (Fig. 4F–I). In mode 3 the dinosaur tracks occur as isolated casts within the mudstone levels (Fig. 4J), revealing the infilling of the footprints produced in the floodplain by passing sands and the later deposition of mudstone.

Tracks preserved as concave hyporeliefs or natural impressions have been described on top of fluvial sandstones (Areny 1, La Llau de la Costa; Fig. 3G and H) and lagoonal limestones (Moror A and B; [18]). In the crevasse splay outcrop of La Llau de la Costa, the footprint preservation depends on the contrast in grain size between successive sedimentation episodes. Thus, track morphology is highly variable throughout the outcrop, probably reflecting trampling at different times.

### Ichnology

**Figure 6:**
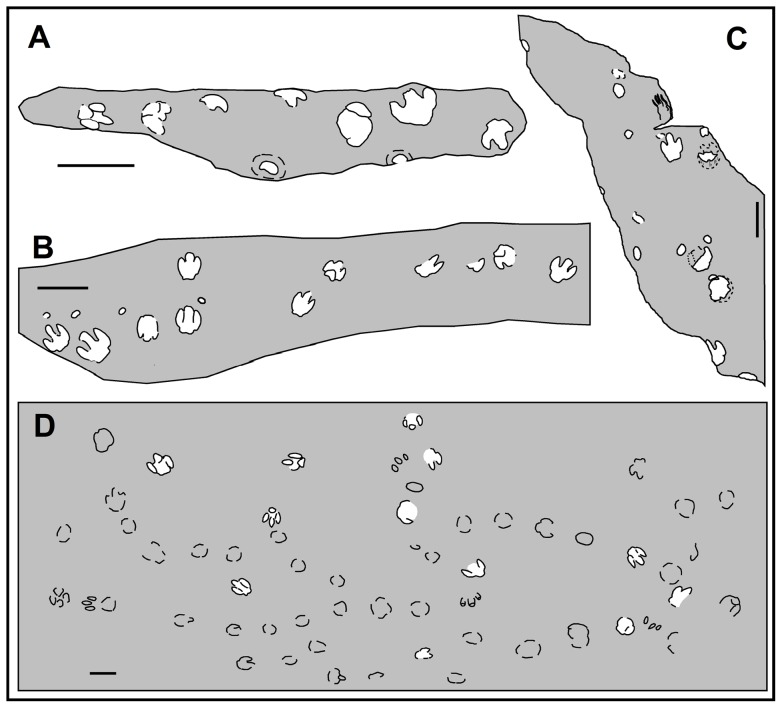
Mapping of some of the main hadrosaur track localities in the Tremp Formation. (A–D) Cingles del Boixader, Masia de Ramon Petjades, La Mata del Viudà, and La Llau de la Costa localities, respectively. Scale bar: 50 cm.

**Figure 7:**
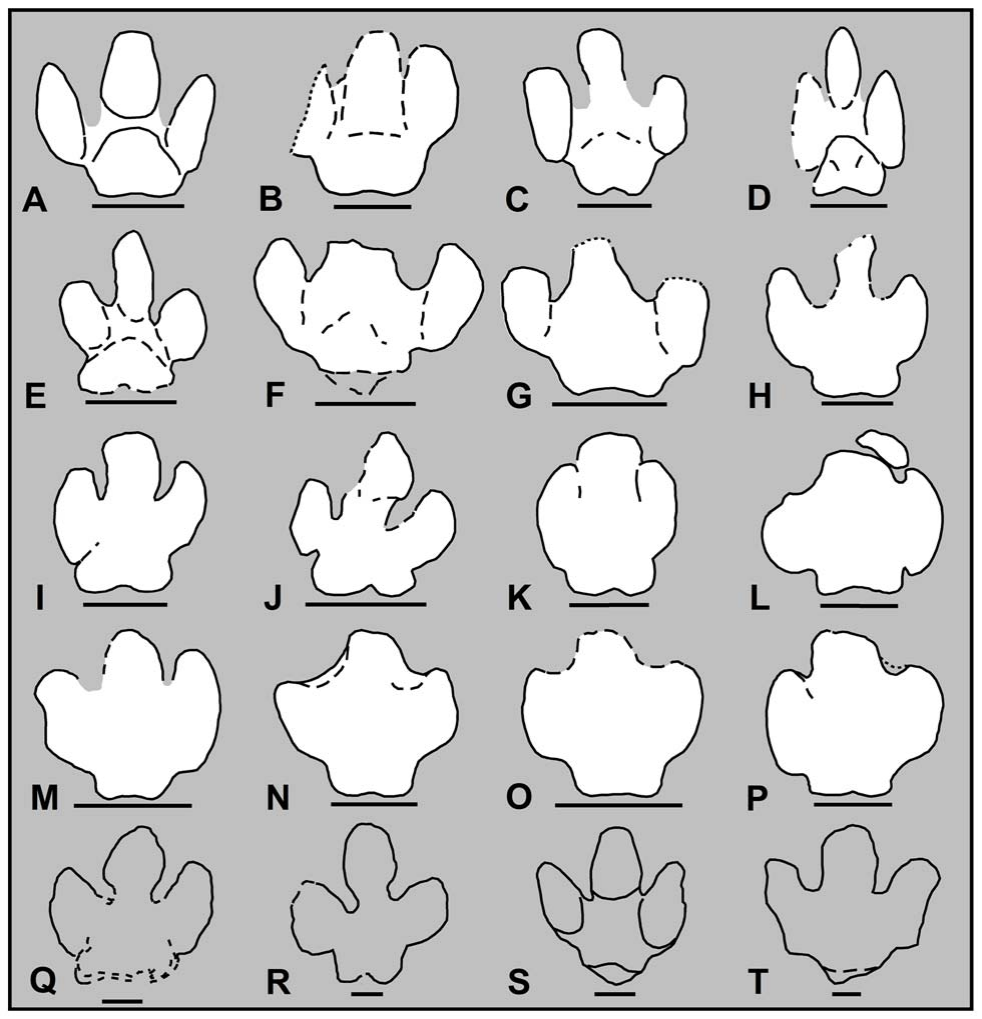
Hadrosaur pedal footprints from the Tremp Formation (white outlines) and other Maastrichtian formations (unfilled outlines) from North America and Asia. (A, C, D) Negative hyporeliefs from the La Llau de la Costa locality (MCD-5140, MCD-5141, MCD-5142, respectively). (B and O) Casts from the Serraduy Norte locality (MPZ 2012/829 and MPZ 2012/827, respectively). (E and F) Casts from the Cingles del Boixader locality (IPS-63661/CB1-CB3). (G) Cast from Serraduy Sur locality (MPZ 2012/828). (H) Cast from the Serrat de Sanguin locality (MCD-5159). (I and K) Casts from the Masia de Ramon Petjades locality (MRP-6, MRP-8). (J) Cast from the Iscles-3 locality (I3–5). (L) Manus-pes casts from the La Mata del Viudà locality (MV-3 and MV-2, respectively). (M and N) Casts from the Camí de les Planes locality (MCD-5149 and MCD-5150, respectively). (P) Cast from the Orcau-4 locality (MCD-5155). (Q) *Hadrosauropodus langstoni* cast (TMP 87.76.6) from the St. Mary River valley locality of the USA, after Lockley et al. [54]). (R) Cast (CU-MWC 224.1–224.11) from the Zerbst ranch locality of the USA, after Lockley et al. [54]. (S) Cast (MPD 100F/11) from the Nemegt locality of Mongolia, after Currie et al. [11]. (T) Cast from the Bugin Tsav locality of Mongolia, after Ishigaki et al. [12]. Scale bar: 15 cm. All drawings are in sole view, except A, C, and D which are in top view.

The new findings in the southern Pyrenees refer to three track types that have been attributed to sauropods (pes) and hadrosaurs (pes and manus) (Fig. 5). The most abundant track types in fluvial settings are the pedal prints of hadrosaurs, which are of moderate sizes and share a similar morphology (Fig. 5, 6, 7 and Appendix S1). These are tridactyl and mesaxonic, as wide as or wider than long (TL: TW ∼1), and have blunt or rounded digits and a broad heel impression. The impression of digit III is thick and equal to or slightly shorter than digits II and IV but protrudes farther anteriorly than these (Fig. 7A, D, E; cf. [52]). The impressions of digits II and IV are sub-equal in length, usually oriented parallel to digit III and have an elliptical to tear-drop shape. Some of the digits preserve hoof-like impressions of unguals (Fig. 5D and 7J). The impression of the heel pad is wide and preserves a bilobed outline (Fig. 5C and E). Laterally, the heel pad displays symmetrical indentations or creases in relation to digits II and IV and constitutes a sub-rectangular morphology (Fig. 7A–P). Some tracks (MCD-5140, MCD-5141, MCD-5156, MRP-10) preserve the morphology of the plantar impression produced by the heel pad (Fig. 5E, 7D, E and Appendix S1), a triangular-shaped area that separates digit III from digits II and IV (i.e. the metatarsophalangeal pad). This is well featured in many other large ornithopod tracks [52]–[54]. Some of the pes casts (at the Serraduy Norte, Serraduy Sur, Serrat de Santó, and La Mata del Viudà localities) preserve slide marks or striae indicating a forward (horizontal and vertical) motion of the foot as it sank into the mud (Fig. 5F). These are vertical on the posterior margin of the heel area and slightly inclined on the lateral/medial sides of the digits. Similar structures have been reported in North American and Asian localities and they have been interpreted as the marks left by the skin tubercles of the foot when it sank into the substrate [11], [53], [55], [56].

The manus tracks are less common in the European record. Those reported in the Pyrenees are smaller and show an oval to sub-rounded morphology without evidence of digital or hoof-like impressions. When associated with the pedal tracks (La Mata del Viudà and Masia de Ramon Petjades sites; Fig. 6B, C and 7L) they are situated anterior and lateral to digit III, between the impressions of digits III and IV, with their long axis oriented somewhat obliquely (about 45°) to the direction of progression. A three-dimensionally preserved manus cast (MCD-5163) from the Serrat de Sanguin locality confirms the ovoid morphology in plantar view and reveals a single, enhanced structure, similar to the mitten described in hadrosaur “mummies” [53]. It preserves vertical slide marks on the anterior margin of the cast.

**Figure 8:**
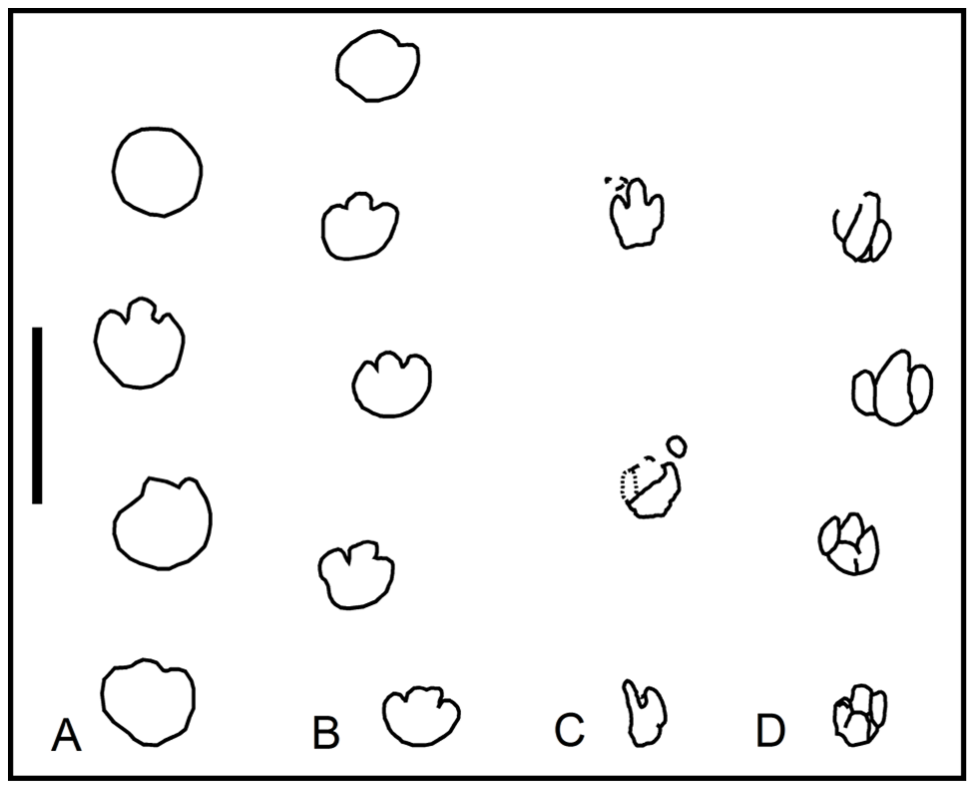
Hadrosaur trackways in southwestern Europe. (A, B, and D) Bipedal trackways from the Areny 1, Moror B, and Sierra de los Gavilanes tracksites (redrawn from Barco et al. [19], Llompart [18], and Herrero-Santos, [24], respectively). (C) Quadrupedal trackway from the La Mata del Viudà tracksite.

Hadrosaur trackways are rare in Europe and mostly show bipedal locomotion (Fig. 8). In bipedal patterns (Fig. 8A, B and D) the pedal tracks are rotated inwards and exhibit a moderately high pace angulation (∼144–166°) and a short stride (SL ∼4.5TL). At the La Mata del Viudà locality, Llompart [17] and López-Martínez et al. [20] suggested that at least three of the pedal tracks were arranged in a trackway but no further measurements or maps were provided. The present study provides a detailed map of the whole surface and the relevant measurements of this trackway (Fig. 8C). The trackway consists of three pedal tracks with corresponding manus tracks, thus indicating a walking hadrosaur with a quadrupedal gait. Notably, the quadrupedal trackway from the La Mata del Viudà locality shows a high pace angulation value (174°) and a long stride (SL∼8TL). Another example of a quadrupedal hadrosaur trackway in the Tremp Formation is at the La Pleta Nord locality, where a sequence of natural casts comprising three large pedes and one manus are aligned to form a trackway (Fig. 3E); the cross-sectional outcrop view prevents descriptions and measurements.

The morphological features of the Tremp Formation tracks (i.e. tridactyl and mesaxonic footprints with broad, blunt digits and a broad heel) are characteristic of large ornithopods [45], [53], [54], [57], [58]. The hadrosaur pedal tracks described in the present study exhibit characteristics that are attributable to the ichnogenus *Hadrosauropodus* [54] on the basis of the following features: (a) tridactyl pes tracks wider than long; (b) blunt, oval digit prints with long axis parallel to track axis; (c) wide bilobed heel; and (d) symmetrical indentations or creases separating the posterior margin of the lateral digits and the heel pad (Fig. 7A–P). They show strong similarities with the hadrosaur tracks described from Maastrichtian deposits of North America and Asia (Fig. 7Q–T; [11], [12], [54]). Conversely, they clearly differ from *Hadrosauropodus nanxiongensis* [59] in the general pedal morphology. These latter tracks, however, are not well preserved [60]. As regards the manus tracks, they are ovoid to sub-rounded in shape and situated between the impressions of digits III and IV, oblique to the direction of progression. This morphology differs from that of *Hadrosauropodus langstoni* in that the latter are sub-triangular in shape but it resembles the morphology described by Currie et al. [53] in a hadrosaur trackway from the Dakota Group of Colorado. It is worth noting that these authors assigned the ichnites to the ichnospecies *Caririchnium leonardii* although the original diagnosis for this ichnospecies [61] included elliptical manus tracks but not a bilobed heel in the hindprints (typical of the ichnogenus *Hadrosauropodus*; [54]). With this in mind, we underscore the necessity of an ichnotaxonomic revision of the Late Cretaceous ornithopod ichnotaxa and propose that the hadrosaur ichnites of the Tremp Formation most probably represent a new *Hadrosauropodus* ichnospecies, different from *H. langstoni* and *H. nanxiongensis*, and with a manus morphology similar to the “*Caririchnium leonardii*” tracks described by Currie et al. [53].

Like other track records of the latest Cretaceous [11], [12], sauropod tracks are less abundant than hadrosaur tracks in fluvial settings. In the Tremp Formation, they include pedal ichnites from Barranc de Guixers-2 and Camí de les Planes, and probably from the Serrat de Sanguin locality, where further excavation is required. In Camí de les Planes and Barranc de Guixers-2 the pedal casts (MCD-5152, MCD-5164 and an uncollected cast, respectively; Dataset S1) are longer than wide (26.5 to 38.5 cm in length) and oval in shape. One of the casts exhibits at least three digital impressions at the anterior margin (Fig. 5G). The sauropod track identified at the Serrat de Sanguin locality is a large and rounded natural cast (∼32 cm across) that may correspond to the infilling of a pedal footprint (Fig. 4B). These all represent the uppermost track record of sauropods (presumably titanosaurs) in Europe, and their distribution is in accordance with the bone record [39].

### Chronostratigraphy

**Figure 9:**
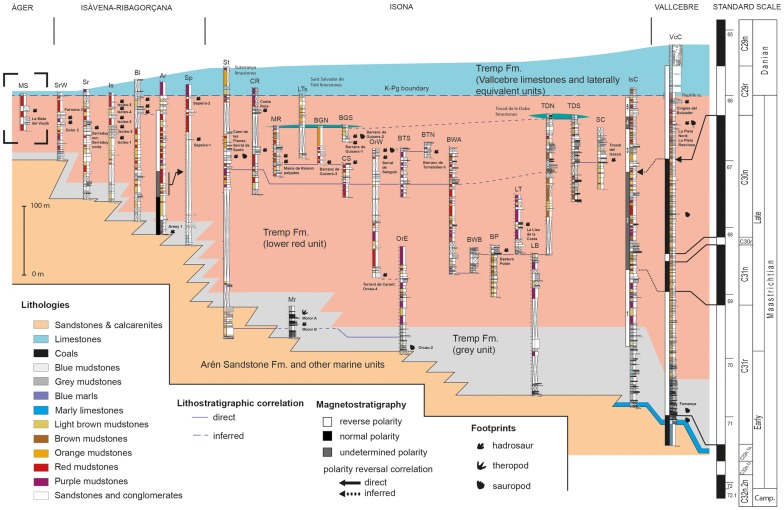
Chronostratigraphic correlation of uppermost Cretaceous deposits (Arén Sandstone and Tremp formations) in the southern Pyrenees (SW Europe). The standard geomagnetic polarity timescale is taken after Ogg and Hinnov [41]. See “Methods” section for further details on section abbreviations and magnetostratigraphical source data.

The integration of the 28 new track localities with the previously known sites and their correlation with the magnetostratigraphic data show a rich succession of dinosaur tracks in the Tremp Formation (Fig. 9 and Table 1). The track succession indicates differences in the temporal distribution of the various dinosaur track types. Hadrosaurs are represented by tracks found noticeably in the late Maastrichtian. The track succession features: (a) an early occurrence of tracks (at the Moror B site) in the upper part of the C31r magnetochron, around the early Maastrichtian–late Maastrichtian boundary (∼70 Ma); (b) a moderate abundance of tracks (at the localities of Areny 1, Basturs Poble, La Llau de la Costa, Torrent de Carant, and Orcau-4) in the C31n-C30r-C30n magnetochrons, in the middle–upper part of the late Maastrichtian (∼69.1–66.3 Ma); (c) a high abundance of tracks in the lower part of the C29r chron (∼66.3–66 Ma), in the latest Maastrichtian. The uppermost unequivocal evidence of hadrosaur tracks in the Tremp Formation occurs at the Cingles del Boixader site, 14 metres below the K-Pg boundary. Other localities with a similarly high chronostratigraphic position are those of Sapeira-2, Iscles-5 and unnamed levels in Blasi (Fig. 9). However, the magnetostratigraphic correlation is still tentative in these sites. Significantly, all the hadrosaur tracks occur in the late Maaastrichtian (at least above the early Maastrichtian–late Maastrichtian boundary), and the highest abundance is found in approximately the last 300,000 years of this time stage. As regards sauropod tracks, they are found from the late Campanian to the latest Maastrichtian and do not show a particular time distribution along the succession. Theropod tracks are scarce in the Tremp Formation and have only been documented at one locality [18].

## Discussion

### Footprint palaeoenvironments and production

The occurrence of dinosaur tracks (mainly and abundantly of hadrosaurs) in continental environments is well documented in various Late Cretaceous (Campanian–Maastrichtian) deposits in North America and Asia [11], [12], [53]–[56]. Even though these do not strictly represent the same sedimentary settings (though most of them correspond to fluvial environments), some authors [53], [55] have underscored their preservational similarities. The lower red unit of the Tremp Formation displays meandering and braided fluvial systems with equally favourable conditions for track production and preservation, similar to those of other fluvial (anastomosed) systems reported in North America and Asia [11], [53], [55], [56]. Our sedimentological data from the Tremp Fm indicate that the braided systems exhibit features shared with the gravel-bed braided rivers of Miall [62]. The meandering systems (and associated crevasses) mainly belong to fine-grained meandering rivers, although some cases may display certain features of the sand-bed meandering rivers of Miall [62]. The close connection with entirely marine deposits through a lagoon is evidenced by regional geology [27]–[29] and by the fact that fine-grained meandering rivers are common in estuarine settings.

**Figure 10:**
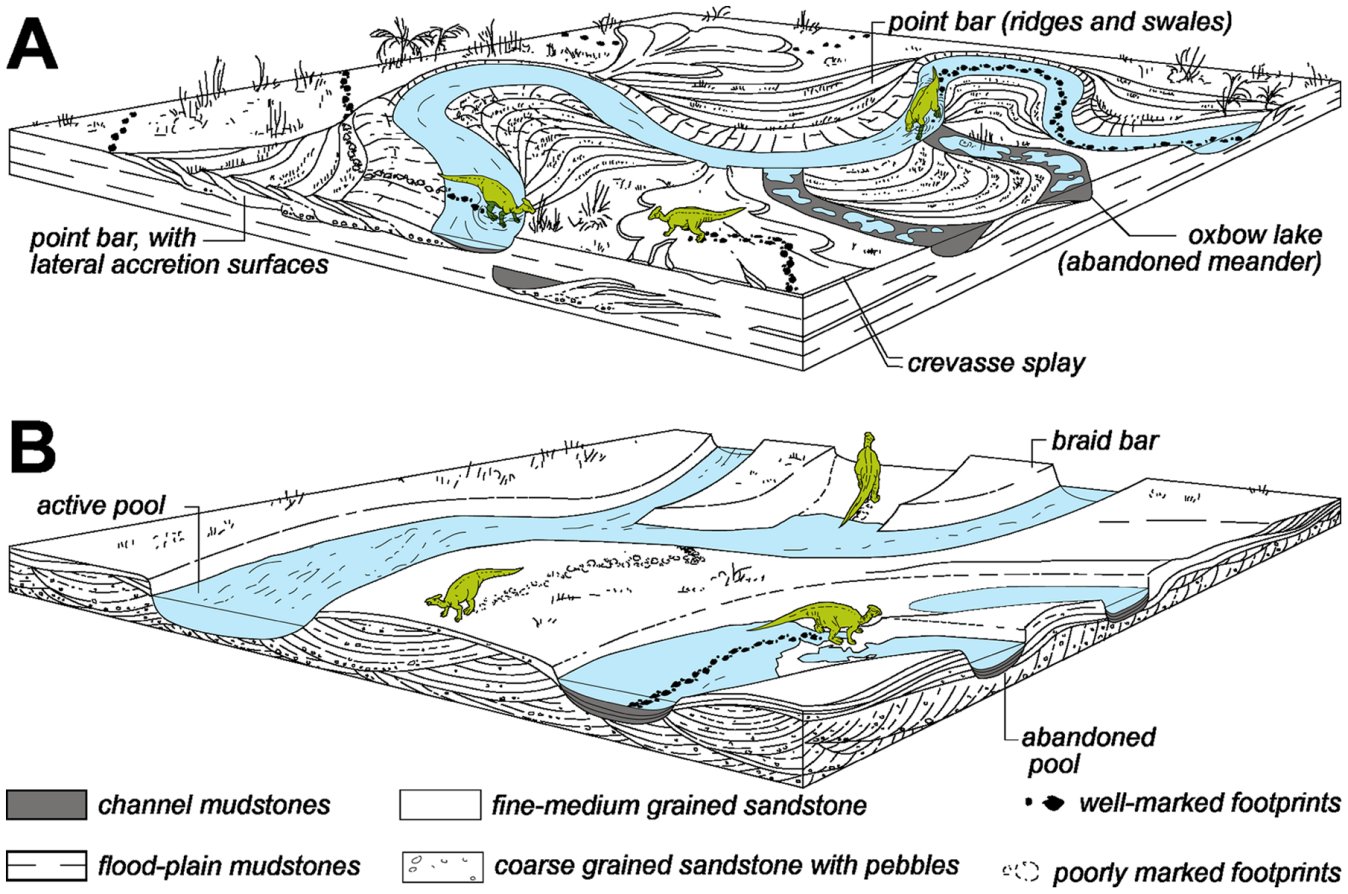
Sedimentary reconstruction of hadrosaur track production in fluvial settings of the Tremp Formation. (A) Sedimentary environments in meandering streams. (B) Sedimentary environments in braided streams.

The preservation mode of footprints as natural casts has been well documented in many Late Cretaceous formations worldwide [11], [12], [52], [55], [56], [63]. The general model for track formation and preservation in fluvial settings highlights that fluctuations in the water table are pivotal for facilitating a suitable substrate [64]. These fluctuations depend on flooding and subsequent emergence episodes, which are related with the hydraulic dynamics of the fluvial channel and probably with seasonal constraints [11], [53]. In the lower red unit of the Tremp Formation the sedimentology of the footprint localities provides data for assessing the production and preservation of the tracks. As in other fluvial settings, the occurrence of tracks preserved as convex hyporeliefs is favoured by the alternating high and low water stages of the fluvial deposits. In the meandering and braided fluvial systems the successive high water stages provided suitable conditions for infilling (sandstone) the footprints produced in the floodplain or in the accretion surfaces within the channel (mudstone). Braided systems are generally less stable than meandering ones, so they have a lower preservation potential for footprints. The dinosaurs produced these tracks on mudstones in low water stage conditions (Fig. 10), and during the high water stage (stream reactivation) the footprints were infilled by sands. The track beds lack evidence of desiccation (e.g. mud-cracks) and this concurs with the hypothesis [11], [56] that the dinosaurs left footprints in wet and muddy substrates in well-drained environments which never dried out and where the water table was close to or above the surface. Some deep casts preserving the three-dimensional shape of the foot (MPZ 2012/826, MPZ 2012/827, IPS 63272, MCD-5154; and Dataset S1) indicate that the water table was probably about 0.2–0.4 m above the surface. Interestingly, some casts (MPZ 2012/826 and MPZ 2012/827; Dataset S1) exhibit burrowing traces, suggesting that invertebrates inhabited the wet sand that infilled the footprint [56]. Extended root mottling and small plant remains in both mudstones and sandstones further indicate that vegetation probably colonized the floodplains, the abandoned channels and the braided and meandering bars, respectively (Fig. 10).

Currie et al. [53] and Nadon [55], [63] pointed out that track formation requires a substrate that is soft enough to be deformed by the animal but firm, cohesive and dewatered enough to retain the shape of the foot until the sediment can infill the cavity. The best-preserved casts in the Tremp Formation reveal the foot shape, thus indicating that the trampled muddy sediment was cohesive enough to resist erosion during the subsequent sheet flood. *Striae* or scratch marks in some of the tracks (Fig. 5F) demonstrate this cohesive feature. By contrast, some badly preserved and deformed casts indicate a less cohesive, softer, muddier substrate that prevented proper preservation of the foot shape (Fig. 4A). In all these cases, the tracks were produced in muddy substrates with the water table close to or above the surface. Conversely, substrates composed of poorly sorted sands with a low portion of cohesive mud (i.e. crevasse splay deposits; Fig. 10B) impede the homogeneous production of tracks, even though some of them are moderately well preserved (Fig. 5A, C).

### Hadrosaur track size

**Figure 11:**
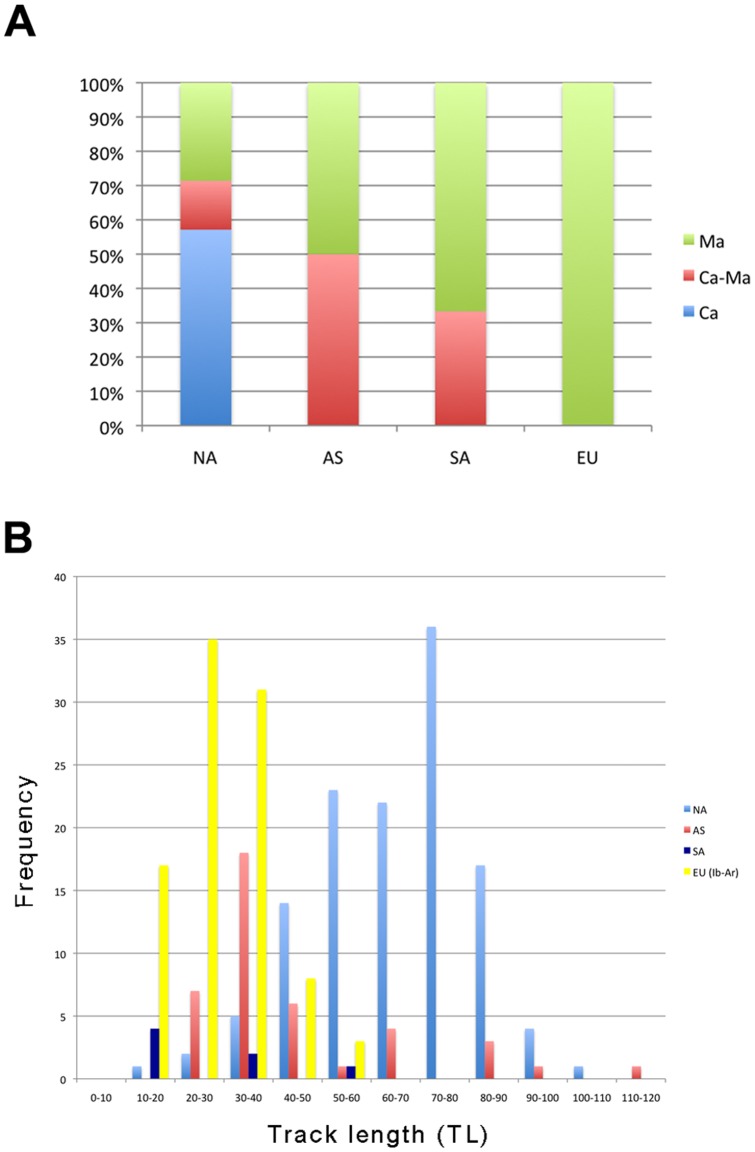
Age distribution and hadrosaur track size. (A) Graph showing the temporal abundance (%) of hadrosaur tracks in geologic formations of North America (NA), Asia (AS), South America (SA), and Europe (EU) through the Campanian, Campanian–Maastrichtian, and Maastrichtian time spans. (B) Size (track length, in cm) distribution of hadrosaur tracks through the abovementioned regions in the Campanian to Maastrichtian time span. Ib-Ar, Ibero-Armorican island. See source data in Dataset S1.

In order to discern biometric and palaeobiogeographic differences and similarities between track makers during the Campanian and Maastrichtian we conducted a quantitative analysis of the size of the tracks attributed to hadrosaur dinosaurs available in the literature (Fig. 11). On the basis of the published data (see Dataset S1), the global record of individual (and measurable) hadrosaur tracks shows that the North American record is composed of individual tracks found in geologic formations of Campanian (57.1%), Campanian–Maastrichtian (14.3%), and Maastrichtian (28.6%) ages. In Asia and South America, the tracks occur in formations of Campanian–Maastrichtian and Maastrichtian age (Asia, Ca–Ma: 50%; Ma: 50%; South America, Ca–Ma: 33.3%; Ma: 66.6%; Fig. 11A). The European record is clearly biased (100% of the samples) in favour of geologic formations of Maastrichtian age, and more particularly, of stratigraphic levels that fall within the C30n and C29r magnetochrons. Southwestern Europe is thus potentially one of the most important areas in terms of yielding terminal Cretaceous track evidence of dinosaurs.

Within Europe, the track record from the Ibero-Armorican island (i.e., one of the ancient paleogeographic regions of the Late Cretaceous European archipelago) is composed of pes tracks that range from 11 to 51.5 cm in length (MCD 2012/831 being the smallest hadrosaur track yet discovered worldwide) and on average (n = 94; mean ± SD = 29±8.9 cm) these tracks are 45% and 65% of the size of those from North America (n = 125; mean ± SD = 64.3±15.6 cm) and Asia (n = 41; mean ± SD = 44.3±20.8 cm), respectively (Fig. 11B). The track record from South America still seems to be too scarce for further conclusions to be drawn but on average the tracks are the smallest in the sample (n = 7; mean ± SD =  26.4±16.1 cm). In the Ibero-Armorican island most of the hadrosauroid genera are of small to moderate size [36], [65], [66]. Thus, the track evidence agrees with data known from the bony record (cf. [46]). The studied sample describes a normal distribution that probably represents the size variability of the trackmakers within a population and this rules out the hypothesis that the Ibero-Armorican track sample may belong to immature juvenile individuals. In consequence, the ichnological data support the hypothesis of the likely influence of insularism on hadrosauroid body size (island rule) in the Ibero-Armorican island [67].

### Biochronostratigraphy

Lockley et al. [9] reviewed the global record of Late Cretaceous dinosaur tracks and underscored the scarcity of the European record. With the new findings of dinosaur tracks in the upper levels of the Tremp Formation of the southern Pyrenees the number of footprint localities in southwestern Europe increases significantly. The track assemblage considered in the present study (with up to 40 track levels) represents the richest and youngest footprint succession in Europe and is among the most complete in the world. Thus, 25 localities have been reported in the C29r magnetochron, very close to the Cretaceous–Palaeogene boundary. The uppermost locality with unequivocal tracks is that of Cingles del Boixader, which is located 14 metres below the K–Pg boundary, in the C29r magnetochron (Fig. 9). This record represents the last dinosaur ichnological occurrence in Eurasia, and one of the latest pieces of evidence for non-avian dinosaurs anywhere in the world.

Furthermore, hadrosaur tracks are significant in terms of the biochronostratigraphy of the Cretaceous landmasses of southwestern Europe. Various authors [68], [69] have hypothesized a faunal turnover within the Maastrichtian in the Ibero-Armorican island characterized by the disappearance of nodosaurids and *Rhabdodon* and the appearance and expansion of hadrosaurs. Currently, the fossil record in Spain and France seems to support this scenario ([70]; but see [71]). Indeed, recent updates of the fossil record and its chronostratigraphic framework in the Tremp basin [6], [46] indicate that hadrosauroid remains are clearly dominant in the late Maastrichtian and yet have not been reported much below the upper part of the C31r, around the early Maastrichtian–late Maastrichtian boundary. Dalla Vecchia et al. [46] suggested that this faunal turnover was due to a time/event-related change rather than an ecological shift. The ichnological data studied here support this interpretation since hadrosaur tracks have been found both in lagoon (e.g. Moror B locality) and fully continental (fluvial) environments, all of late Maastrichtian age, though no hadrosaur tracks have been found in similar environments much below the early Maastrichtian–late Maastrichtian boundary. As most of them are found in a distinct temporal distribution within the late Maastrichtian, they show a specific time-span distribution. Therefore, the occurrence of hadrosaur tracks in the Ibero-Armorican island seems to be indicative of a late Maastrichtian age, and these tracks are thus important biochronostratigraphic markers in the faunal successions of the Late Cretaceous of southwestern Europe. As regards sauropods, the present data confirm that they were present at the very end of the Maastrichtian (Fig. 9), as indicated by the body fossils [39].

## Conclusions

Recent findings in the Tremp Formation (southern Pyrenees, SW Europe) reveal that dinosaur tracks are much more abundant than previously thought. Sedimentological, ichnological, and chronostratigraphic analyses highlight the following conclusions:

- The fluvial lower red unit of the Tremp Formation exhibits meandering and braided fluvial systems with favourable conditions for track production and preservation, like those of North America and Asia.
- The dinosaurs mainly produced the tracks on the floodplain, within the channels, and on and within crevasse splay deposits in low water stage conditions, and the footprints were infilled by sands during high water stage (stream reactivation).
- The track record is composed of abundant hadrosaur and scarce sauropod and theropod tracks. The hadrosaur tracks are significantly smaller in size but morphologically similar to comparable records in North America and Asia. They are attributable to the ichnogenus *Hadrosauropodus*.
- A rich track succession composed of more than 40 distinct track levels indicates that hadrosaur footprints are found above the early Maastrichtian–late Maastrichtian boundary and most noticeably in the late Maaastrichtian, with tracks occurring abundantly in the Mesozoic part of the C29r magnetochron, in the latest 300,000 years of the Cretaceous.
- The occurrence of hadrosaur tracks in the Ibero-Armorican island seems to be characteristic of the late Maastrichtian time interval and thus they are important biochronostratigraphic markers in the faunal successions of the Late Cretaceous in SW Europe.

## Supporting Information

Text S1 **Brief description of the main track localities found in the southern Pyrenees.**
(DOCX)

Dataset S1 **Measurements (in cm) taken for the latest Cretaceous (Campanian–Maastrichtian) hadrosaur pedal tracks reported in the literature.** Abbreviations: NA: North America; AS: Asia, SA: South America; EU: Europe; Ca: Campanian; Ca–Ma: Campanian–Maastrichtian; Ma: Maastrichtian; TL: track length; TW: track width.
(XLSX)

Appendix S1 **Photogrammetric models of the hadrosaur pes MCD-5140 and MCD-5142 from** **the La Llau de la Costa tracksite. Scale bar: 15 cm.**
(ZIP)
