# Supplementary material for: The Latest Succession of Dinosaur Tracksites in Europe: Hadrosaur Ichnology, Track Production and Palaeoenvironments
Source: PLoS One. 2013 Sep 3;8(9):e72579. doi: 10.1371/journal.pone.0072579 (PMC3760888; doi:10.1371/journal.pone.0072579)
Supplement: Text S1 — Brief description of the main track localities found in the southern Pyrenees. (DOCX) [file pone.0072579.s001.docx]

**Text S1 Brief description of the main track localities found in the southern Pyrenees.**

The following descriptions refer to the new track localities as well as other previously known sites reported in the literature. We use the stratigraphic distance between the localities and the base of the Vallcebre limestones and lateral equivalent strata as an indicator of site proximity with respect to the Cretaceous–Palaeogene boundary (see main text).

*Vallcebre Syncline sector*

**Cingles del Boixader** - The site is found in the upper part of the lower red unit (“Gres à reptiles” member) of the Tremp Formation, 14 meters below the Cretaceous–Palaeogene boundary (in the lower half of the C29r magnetochron). The site consists of a 5 meters-long overhanging ledge that contains nine tracks preserved as natural casts at the base of a sandstone level (Fig. 3B, 6A). Seven tracks are attributed to pedal (see measurements in Dataset S1) and two to manus footprints (TL=8 and 10 cm; TW= 13,5 and 13 cm, respectively) of hadrosaurs; two partial impressions of pedal digits are also distinguishable. All the tracks were replicated and catalogued (IPS-63661).

**La Pleta Nord** and **La Pleta Resclosa** - The two sites are found in the lower red unit, about 40 meters below the Cretaceous–Palaeogene boundary, within the C29r magnetochron. They are probably stratigraphically equivalent and are located some hundred meters apart from one another. The La Pleta Nord site contains several track casts and various track-like load structures exposed in cross-sectional outcrop views (Fig. 4C and G). Three casts probably correspond to pedal tracks of hadrosaurs and are aligned to form a short trackway (Fig. 3E). There is a manus cast in front of one of these tracks. A small hadrosaur pedal track was also documented at the site (see measurements in Dataset S1). The La Pleta Resclosa site contains two tracks preserved as natural casts (convex hyporeliefs) at the base of a thin sandstone level with multiple accretion surfaces. They are stacked vertically and are viewed in cross-section (Fig. 4H). One of the tracks may resemble a small sauropod pes as it presents various lateral indentations that may correspond to digital traces, but further excavation is required. The other track is of uncertain affinity.

**Fumanya tracksites** – The Fumanya localities are located in the lowermost levels of the grey unit of the Tremp Formation, in the early Maastrichtian (C32n.1n) [31]. They consist of extensive and vertical exposures of marly limestones bearing about 3,000 sauropod (titanosaur) tracks and 55 trackways [13, 49]. The tracks occur as concave hyporeliefs in at least four distinct stratigraphic levels, which record the transition from marine to lagoon environments [51].

*Eastern Tremp Syncline (Isona sector)*

**La Llau de la Costa** - The locality is located in the middle part of the lower red unit of the Tremp Formation, 185 meters below the Cretaceous–Palaeogene boundary, probably in the lower part of the C30n magnetochron (late Maastrichtian). The site consists of 140m^2^ horizontal surface with about 50 tracks (concave hyporeliefs). Most of them are badly preserved (Fig. 3H) due to present erosion or most probably to original substrate conditions during track production; however, about ten tracks exhibit good morphological details and four have been replicated (MCD-5140, MCD-5141, MCD-5142, MCD-5143; Figure 5A, C, and Appendix S1) and measured (see measurements in Dataset S1). They all belong to hadrosaur pes and some of them may align in trackways. MCD-5140 is the best preserved, exhibiting exemplary features of the Tremp Formation hadrosaur pes prints (Fig. 5A, 7A); MCD-5142 displays a characteristic bilobed outline in the posterior heel margin (Fig. 5C, Appendix S1). The tracks are impressed on multiple trampling surfaces of poorly sorted grey sandstones representing the crevasse splay facies of a meandering stream.

**Masia de Ramon Petjades** - The site is found in the lower red unit, 100 meters below the Cretaceous–Palaeogene boundary, in the lower part of the C29r magnetochron. The outcrop contains 14 hadrosaur tracks preserved as natural casts (convex hyporeliefs) underneath an overhanging ledge of sandstone ledge. Most of the tracks correspond to pedal footprints (see measurements in Dataset S1) and only three tracks represent manus prints (TL=5.5 cm; TW=8.5 cm). The tracks are distributed randomly without a preferential direction and no trackway arrangement is distinguished (Fig. 6B). Two pedal cast tracks were collected as isolated blocks from the same channel body and catalogued in the Museu de la Conca Dellà collection (MCD-5156 and MCD-5157).

**Barranc de Guixers-1**, **Barranc de Guixers-2**, and **Barranc de Guixers-3** – These sites correspond to three close stratigraphic levels found close to one another in the lower red unit of the Tremp Formation, 65, 60 and 98 meters below the K–Pg boundary (C29r magnetochron), respectively. Barranc de Guixers-1 used to contain at least two hadrosaur pes tracks preserved as natural casts at the bottom of a sandstone layer (recently the overhanging ledge collapsed and the tracks vanished). Barranc de Guixers-2 is a small outcrop containing a hadrosaur pes track (uncollected) and two likely sauropod pes casts (MCD-5164 and another uncollected sample), as well as multiple scratches attributed to other reptiles. The Barranc de Guixers-3 outcrops contain various hadrosaur tracks preserved as natural casts (MCD-5153) at the bottom of sandstone layers.

**Camí de les Planes**, and **Serrat de Santó** – These localities (also named “Suterranya”) and other unnamed track levels west of the village of Suterranya are found in the upper part of the lower red unit of the Tremp Formation (104 meters below the K–Pg boundary, in the C29r magnetochron). They are equivalent stratigraphically equivalent to one another and contain abundant hadrosaur and (less abundantly) sauropod tracks preserved as natural casts (convex hyporeliefs). The tracks are usually found isolated as sandstone blocks that have come off the cross-sectional outcrop. MCD-5154 from the Serrat de Santó site is a three-dimensional cast of a hadrosaur pes, preserving striae and allowing inferences on the locomotor mechanics of the trackmaker. Ten tracks have been collected from both localities (see Dataset S1).

**Serrat de Sanguin** – The locality of Serrat de Sanguin is found in the upper part of the lower red unit of the Tremp Formation, 81 meters below the Cretaceous–Palaeogene boundary (C29r magnetochron). Dinosaur tracks, mostly hadrosaur footprints, occur as natural casts (convex hyporeliefs) at the base and within various levels of fluvial sandstone. Most of the tracks represent tridactyl pedal tracks of hadrosaurs (MCD-5159, MCD-5161, MCD-5162) though a three-dimensionally preserved manus cast was also collected (MCD-5163). A large, rounded cast was observed at the outcrop and this may belong to a sauropod footprint (Fig. 4B).

**Tossal del Gassó, Costa de la Serra-4, Orcau-4** (=Barranc de Torrebilles-8), **Basturs Poble, Torrent de Carant**, **Barranc de Torrebilles-5, Costa Roia**, and other unnamed track levels – The localities are found in the middle and upper part of the lower red unit of the Tremp Formation, within the Maastrichtian part of the C29r magnetochron, a few dozen meters below the Cretaceous–Palaeogene boundary (except for Torrent de Carant and Orcau-4 (C31r), and Basturs Poble (C31n or C30r)). Most of the sites preserve several track-like load structures as well as well-defined hadrosaur pedal casts underneath overhanging sandstone ledges. Some of the collected tracks are good examples of the pedal morphology of Tremp Formation hadrosaurs (e.g. MCD-5155, MCD-5166; Fig. 7). An uncollected block near the Masia de Ramon petjades site preserves two pedal track casts of hadrosaurs (see measurements in Dataset S1)

**Moror A** and **Moror B** – The localities are found in the grey unit of the Tremp Formation around the early Maastrichtian–late Maastrichtian boundary (middle part of the C31r magnetochron). In Moror A a dozen of tracks are exposed in a small outcrop with a surface of about 35m^2^. They are preserved as concave epireliefs, are tridactyl in shape, and have been attributed to theropods [18]. Moror B consists of 50-225m^2^ surface of bioturbated grey limestone preserving several isolated tracks and one trackway attributed to hadrosaurs [18].

**Orcau-2** – The locality is located in the grey unit of the Tremp Formation, around the early Maastrichtian–late Maastrichtian boundary (lower part of the C31r magnetochron), and has been characterized as lagoonal [6]. The tracks are preserved as concave hyporeliefs on a sub-vertical limestone exposure with abundant burrowing. Llompart et al. [14] first reported the locality with the description of the ichnogenera *Ornithopodichnites* and *Orcauichnites* (considered *nomina dubia* by Lockley and Meyer [22]) and sauropod footprints. A recent assessment of the site has provided evidence of titanosaur trackways [15].

*Western Tremp Syncline (Isàvena-Ribagorça sector)*

**Sapeira-1** and **Sapeira-2** - The localities of Sapeira are found in the upper part of the lower red unit of the Tremp Formation, tentatively about 64 and 5 meters below the Cretaceous–Palaeogene boundary (C29r magnetochron), respectively. Each site preserves one hadrosaur pes track at the bottom of red, fine-grained sandstones. In Sapeira-1 the track (IPS-63272) is preserved three-dimensionally, with a tridactyl appearance (Fig. 4D).

**Areny 1** - The site is found in the grey unit of the Tremp Formation, at the base of the C30n magnetochron [35]. It consists of a 55m^2^ surface containing about 20 tracks preserved as concave epireliefs and attributable to hadrosaurs, some of them arranged in two (or probably three) trackways [19].

**Iscles-1, Iscles-2, Iscles-3**, **Iscles-4**, and **Iscles-5** – The Iscles localities are found in the lower red unit of the Tremp Formation, within the Maastrichtian part of the C29r magnetochron. Iscles-1 (60 meters below the K–Pg boundary) consists of a small, vertical exposure of sandstone that corresponds to an accretion surface of a meandering fluvial channel. The surface contains two casts (convex hyporeliefs) of circular shape that probably correspond to pedal footprints of hadrosaurs. An additional cast of a hadrosaur pes print was collected and catalogued in the Museo Paleontológico de Zaragoza (MPZ 2012/831). This is the smallest hadrosaur pes track reported worldwide. Iscles-2 is found 42 meters below the Cretaceous–Palaeogene boundary. It consists of a small outcrop, which bears various convex hyporeliefs at the bottom of a sandstone body. Iscles-3 (33 m below the K-Pg boundary) displays tracks preserved as natural casts (convex hyporeliefs) at the base of the successive accretion surfaces and exposed in vertical overhanging ledges (Fig. 4A). The four track surfaces contain a total of 40 tracks including manus and pes, attributable to hadrosaurs of various sizes (see measurements in Dataset S1); some of them may be aligned to form trackways. One track (MPZ 2012/830) was collected and catalogued in the Museo Paleontológico de Zaragoza. Iscles-4 and Iscles-5 are located 17 and 10 meters below the Cretaceous–Palaeogene boundary, respectively. The former site contains one single, vertically stacked and three-dimensionally preserved track; the latter preserves a single natural cast (convex hyporelief). Both footprints have been attributed to hadrosaur pes prints.

**Serraduy Sur** and **Serraduy Norte** – The Serraduy localities are found in the lower red unit of the Tremp Formation, 46 meters below the Cretaceous–Palaeogene boundary (C29r magnetochron). They are stratigraphically equivalent to one another and preserve similar tracks referable to hadrosaurs. The Serraduy Sur locality (172-J-04-A level; Fig. 3F) preserves various tracks, including various pes (see measurements in Dataset S1) and one manus cast (MPZ 2012/833; TL=12 cm; TW=10 cm). Well-preserved, three-dimensional casts of hadrosaur pes (MPZ 2012/826 and MPZ 2012/827) preserve *striae* and allow inferences on the locomotor mechanics of the trackmaker. The Serraduy Norte locality (172-I/12/H level) consists of three track horizons that constitute the architecture of a meandering fluvial channel. At least a ten of (pedal) tracks preserved as natural casts (convex hyporeliefs) occur at the base of each accretion level (Fig. 4F).

**Dolor 2**, **Fornons 3** and other unnamed track levels in the Blasi section – These outcrops are found in the upper part of the lower red unit of the Tremp Formation, about 10-40 meters below the Cretaceous–Palaeogene boundary (C29r magnetochron). They exhibit various several track-like load structures, exposed underneath overhanging sandstone ledges or in cross-sectional outcrop views.

*Àger Syncline sector*

**La Mata del Viudà** – The locality (also named “Corçà”, “Mas de Saurí” or “Millà”) is located in the uppermost part of the lower red unit of the Tremp Formation, about 25 meters below the Cretaceous–Palaeogene boundary, at the base of the C29r magnetochron [72]. The tracks are preserved as natural casts (convex hyporeliefs) in a 5m^2^-overhanging ledge at the bottom of a sandstone layer (Fig. 3C). Llompart [17] distinguished about 20 tracks, mostly attributed to ornithopod dinosaurs, and also noted the occurrence of traces referable to other reptiles. The present study provides a complete mapping of the site (Fig. 6C) and describes and measures a likely trackway (Fig. 8C) and the morphologies of particular pes and manus tracks (Fig. 7L). The pes (n=5; mean values TL=32 cm; TW=32.6 cm; see further measurements in Dataset S1) and manus tracks (n=6; TL=11 cm; TW=13 cm) have been attributed to hadrosaurs. In 2010 part of the overhanging surface collapsed and five of the tracks (MV10, MV11, MV12, MV13, MV14) vanished.

**La Massana** – The locality (also named “Camarasa”) is located in the lower levels of the grey unit of the Tremp Formation, in the late Campanian [50]. It consists of 1,200 m^2^ of limestones exposures bearing an estimate of 900 tracks attributed to sauropods [16]. The tracks are rounded in shape and are preserved as concave hyporeliefs; no discrete trackways have been distinguished.

**Rereferences**

1. López-Martínez N, Fernández-Marrón MT, Valle MF (1999) The succession of vertebrates and plants across the Cretaceous-Tertiary boundary in the Tremp Formation, Àger valley (South-central Pyrenees, Spain). Geobios 32: 617–627.
